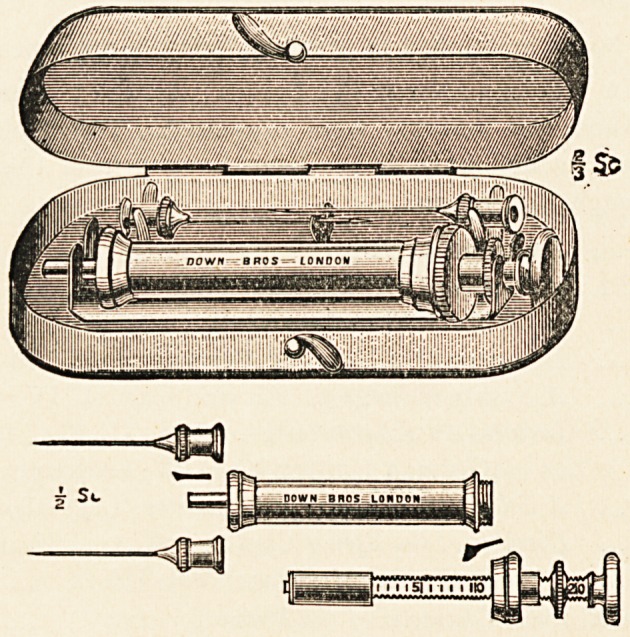# Notes on Preparations for the Sick

**Published:** 1897-09

**Authors:** 


### Notes on Preparations for the Sick.

**Tannalbin-Knoll.**—B. Kühn, London.—This is an albuminate of tannin; it is a pale yellow powder, tasteless, which resists the action of gastric juice, but decomposes slowly in the intestine. A dose of one gramme, three or four times a day, has been of use in cases of diarrhœa.

**Tabloids: Ox Bile,** gr. iv.; **Salivary Gland Substance,** gr. v.; **Liver Substance,** gr. v.; **Kidney Substance,** gr. v.; **Fallopian Tube Substance,** gr. v.; **Spinal Cord Substance,** gr. iiss.; **Cervical Lymphatic Gland Substance,** gr. iij.; **Pineal Gland,** gr. j.—BURROUGHS, WELLCOME & Co., London.—Many other glands and organic substances are laid under contribution by this firm to supply them with tabloids. It appears to be assumed that every tissue and organ of the body furnishes an internal secretion which exercises an important influence upon the process of metabolism, and hence it is inferred that small doses of those tissues and organs given in the form of a tabloid made up of the dried substance of the tissues or organ, containing therefore the substance itself of the gland or tissue, and having within it all the active constituents of the tissue concerned, must be of value in the treatment of many diseases.

Of the uses of the thyroid in myxœdema, and of the uses of blood serum as an antitoxin in diphtheria, we need no longer have any hesitation or doubt; but when it is assumed that because of the results in those two instances we may infer like results from many other organic bodies, we have yet to ask for the evidence in favour of their utility.

We cannot but suspect that in the case of many of these substances the mystic and the mythical are being allowed to serve for evidence. Surely in the theories underlying the administration of these substances we have dreams of a wilder character than any of those which have been imputed to Hahnemann.

---

**Pure Cod Liver Oil.**—PETER MÖLLER, London.—By many people the mere suggestion of cod liver oil is at once declined with something resembling disgust, and no wonder when the patient has been deluged with the stronger and much flavoured as well as malodorous varieties of the oil of the Gadus Morrhua, charged with decomposition products of albumen which are derived from the putrefaction of liver tissues and by the oxidation of free fatty acids.

By Peter Möller's method the livers are used while fresh, and the oil is extracted by steam, every stage of the manufacture being conducted under an atmosphere of carbonic acid gas. The product is colourless, odourless, and tasteless, and is free from the decomposition products of both albumens and fats. Ninety-six per cent. of this oil consists of glycerides, peculiar to cod liver oil: they are the so-called "active principle," which are extracted "pure and simple," and have to be protected from their special liability to oxidation. It is interesting and important to know that any other "active principles" are impurities with which the oil has been contaminated by the putrefaction of the liver and the subsequent oxidation of the extract: these are mostly ptomaïnes, and are more or less powerful poisons. (See *Cod Liver Oil and Chemistry*, which we reviewed in September, 1895.)

**Trommer's Extract of Malt with Cod Liver Oil. Trommer's Extract of Malt with Hypophosphites.**—The Trommer Extract of Malt Company, Fremont, Ohio.—Messrs. Francis Newbery and Sons have sent us samples of these preparations which have obtained a considerable degree of popularity. Experiment shews that they have a high degree of diastasic activity, and they are as palatable as such sticky preparations can be made.

---

**Keros.**—Bouillon Fleet Limited, London.—There seems to be no end to the varieties of fluid beef. This is a viscid fluid, which after it is diluted to 1 in 10 shows a specific gravity of 1040. The analysis of Dr. A. B. Griffiths, F.C.S., gives the following results:—

| | | | |
|---|---|---|---|
| Moisture ... ... ... | 15.00 | Albumen ... ... | 26.24 |
| Fibrine ... ... ... | 40.06 | Salts... ... ... | 18.70 |

The fluid gives an abundant precipitation with heat and nitric acid; also with picric acid it amounts to 1 per cent. after diluting to 1 in 10. It contains much hæmoglobin, and under the microscope shews a considerable quantity of starch granules distorted by heat, which appear to be from either wheat or barley, probably the latter. Why should this be? It is a highly nutritive fluid, and is more palatable than most meat extracts; but the starch does not appear in the analysis, and its presence in quantity perhaps accounts for the apparent over estimate of the amount of albuminous matter and fibrine.

---

**Liebig's Extract of Meat and Malt Wine.**—Stephen Smith and Co., London.—The *Lancet* analysis shows: Alcohol, 12.62 per cent.; extractives, 12.84 per cent.; mineral matter, 0.77 per cent. Of the extractives it was found that 1.22 parts consisted of nitrogenous principles. It is a pleasant fluid to drink, and must have a considerable value as a stimulant, as well as a minor degree of tissue-building nutrient utility.

---

**L L Whisky.**—Kinahan & Co., Dublin.—In order to secure a permanent supply, his Grace the Lord Lieutenant of Ireland in the year 1807 ordered that the whole of a large vat containing the spirit should be reserved for his use. The letters "L L, Lord Lieutenant, with coronet over, were forthwith painted on the vat and this particular description of spirit has ever since been known under the above designation. It is of one uniform quality, and is bottled exclusively by Messrs. Kinahan & Co. The spirit has an excellent flavour and aroma, it contains practically no extractives, and its acidity is almost *nil*.

**Metal Aseptic Hypodermic Syringe.**—DOWN BROS., London.—The advantage of having in a hypodermic syringe a piston rod made of some permanent material which is not liable to get out of order, and is also capable of sterilisation by boiling, is at once apparent. In this syringe a metal barrel is substituted for the usual one of glass, which admits of being ground accurately inside to fit the piston rod, and works with perfect smoothness when lubricated with vaseline. An additional advantage in the metal barrel is that it is not liable to breakage, and that it can be boiled without fear of detaching the mounts. The syringe is supplied with either steel or platinum iridium needles, and put up in a neat metal case.

## Figures and Tables

**Figure f1:**